# **“**The new normal has become a nonstop crisis”: a qualitative study of burnout among Philadelphia’s harm reduction and substance use disorder treatment workers during the COVID-19 pandemic

**DOI:** 10.1186/s12954-023-00752-7

**Published:** 2023-03-11

**Authors:** Ijeoma C. Unachukwu, Matthew P. Abrams, Abby Dolan, Kehinde Oyekemi, Zachary F. Meisel, Eugenia C. South, Shoshana V. Aronowitz

**Affiliations:** 1 Rutgers Robert Wood Johnson Medical School, New Brunswick, NJ USA; 2 Urban Health Lab, University of Pennsylvania, Philadelphia, PA USA; 3 Center for Emergency Care Policy and Research, University of Pennsylvania, Philadelphia, PA USA; 4 University of Pennsylvania School of Nursing, Philadelphia, PA USA

## Abstract

**Background:**

The COVID-19 pandemic worsened the ongoing overdose crisis in the United States (US) and caused significant mental health strain and burnout among health care workers (HCW). Harm reduction, overdose prevention, and substance use disorder (SUD) workers may be especially impacted due to underfunding, resources shortages, and chaotic working environments. Existing research on HCW burnout primarily focuses on licensed HCWs in traditional environments and fails to account for the unique experiences of harm reduction workers, community organizers, and SUD treatment clinicians.

**Methods:**

We conducted a qualitative secondary analysis descriptive study of 30 Philadelphia-based harm reduction workers, community organizers, and SUD treatment clinicians about their experiences working in their roles during the COVID-19 pandemic in July–August 2020. Our analysis was guided by Shanafelt and Noseworthy’s model of key drivers of burnout and engagement. We aimed to assess the applicability of this model to the experiences of SUD and harm reduction workers in non-traditional settings.

**Results:**

We deductively coded our data in alignment with Shanafelt and Noseworthy’s key drivers of burnout and engagement: (1) workload and job demands, (2) meaning in work, (3) control and flexibility, (4) work-life integration, (5) organizational culture and values, (6) efficiency and resources and (7) social support and community at work. While Shanafelt and Noseworthy’s model broadly encompassed the experiences of our participants, it did not fully account for their concerns about safety at work, lack of control over the work environment, and experiences of task-shifting.

**Conclusions:**

Burnout among healthcare providers is receiving increasing attention nationally. Much of this coverage and the existing research have focused on workers in traditional healthcare spaces and often do not consider the experiences of community-based SUD treatment, overdose prevention, and harm reduction providers. Our findings indicate a gap in existing frameworks for burnout and a need for models that encompass the full range of the harm reduction, overdose prevention, and SUD treatment workforce. As the US overdose crisis continues, it is vital that we address and mitigate experiences of burnout among harm reduction workers, community organizers, and SUD treatment clinicians to protect their wellbeing and to ensure the sustainability of their invaluable work.

## Introduction

Drug overdoses across the United States (US) have risen threefold over the past 20 years, and overdose is now the leading cause of injury-related death [1, 2]. Despite this, community-based efforts to address this overdose crisis, including evidence-based substance use disorder (SUD) treatment and harm reduction services, are woefully underfunded and under-resourced [3]. The COVID-19 pandemic has exacerbated underlying structural causes of the overdose crisis and created additional challenges for people who use drugs (PWUD) [4, 5], including instability and contamination of the street drug supply [6], reduced access to harm reduction services and SUD treatment; [4, 7, 8], isolation and financial stressors leading to higher rates of drug use and relapse [9, 10], and a focus of emergency departments on COVID-19 patients rather than patients with SUD [11]. In light of the COVID-19 pandemic, SUD-related morbidity and mortality continue to increase and are projected to continue [12].

### Overdose crisis in Philadelphia

Philadelphia, the site of this study, has the second-highest overdose mortality rate across all US counties. [13, 14] Following the onset of the COVID-19 pandemic, the rate of fatal overdoses in Philadelphia rose 5.5% from the year before [13]. More than 80% of deaths involved fentanyl [13]. As in many other parts of the US, Philadelphia’s street drug supply has been flooded with fentanyl-contaminated products including pills made to appear like stimulants, oxycodone, and benzodiazepines, and the “heroin” supply is increasingly more fentanyl than heroin [15].

Philadelphia also faces a housing security crisis with nearly 6,000 Philadelphians lacking secure and stable housing [16]. Most deaths among Philadelphians experiencing homelessness in 2019 were caused by drug overdoses, primarily opioids [17]. Philadelphia neighborhoods with high rates of drug use, like the Kensington neighborhood, also have higher rates of police surveillance [18]. Fear of legal repercussion and maltreatment by law enforcement or medical professionals have caused reluctance on the part of bystanders and people who use drugs to call for emergency services in situations where someone may be overdosing which in turn can increase the likelihood of mortality [19]. Some Pennsylvania state laws may still even allow for the arrest and punishment of those who provide substances to someone who suffers a fatal overdose (drug-induced homicide laws), which can discourage alerting emergency services in overdose situations [19, 20].

In Philadelphia and across the world, the COVID-19 pandemic created changes in the delivery of community-based programs and harm reduction services [21]. Restrictions such as travel restrictions, social distancing guidelines, and the shutdown on non-essential services caused a disruption of routine services [21]. While telehealth was used to compensate for gaps in care, its success was not consistent across communities [21]. Creative solutions, such as the development of mobile units, to continue needle exchange programs of methadone clinics also developed during this time [21]. Philadelphia addresses overdose prevention is addressed by a mix of housing, harm reduction, and SUD treatment groups and organizations. While some efforts to treat SUD and prevent overdose are carried out by traditional healthcare workers, many harm reduction efforts and services are spearheaded by volunteers and community organizers, many of whom have lived experience of substance use, SUD, and/or housing insecurity [22–24]. Individuals with lived experience often have a better understanding of what is happening in the everyday lives of PWUD and people experiencing unstable housing and can promote trust between marginalized individuals and traditional healthcare and human services organizations. This work is central to overdose prevention efforts in the city.

### Burnout among healthcare workers

Burnout among healthcare workers has been a rising health concern in recent years [25, 26]. In healthcare and psychology, “burnout” refers to negative psychological symptoms caused by long-term exposure to chronic workplace stressors, including overwhelming exhaustion, a sense of ineffectiveness, lack of accomplishment, or uncertainty about the future [25]. Reasons for the rise in burnout prevalence among healthcare workers include long working hours, insufficient compensation, lack of clinical autonomy, and increasing computerization of practice [27]. Burnout is experienced by many workers in people-oriented ‘caring’ health-related professions, particularly those whose roles pertain to trauma or stressful conditions [26, 28]. Symptoms of burnout including depression, energy depletion, and anxiety are on the rise across the healthcare workforce [29]. They have repercussions for the public such as negative patient outcomes [30–32] and higher healthcare costs [33, 34].

The COVID-19 pandemic worsened mental health strain and healthcare-related burnout among health care workers [35–45]. Contributing to the strain were rapid shifts in COVID-19 case volume, critical supply shortages (e.g., personal protective equipment), lagging vaccination rates, and evolving public health guidelines [43, 46–48]. The tremendous growth of mental health strain and burnout among healthcare workers in hospital settings has received national attention. For example, President Biden recently signed the Dr. Lorna Breen Health Care Provider Protection Act, legislation allotting resources for burnout and suicide prevention among healthcare workers, inspired by Dr. Breen’s death by suicide from the strain of providing emergency department care during the COVID-19 pandemic [49].

Most of the national attention surrounding healthcare worker mental health is focused on burnout among licensed workers in traditional healthcare environments like hospitals [35–45]. However, community-based providers of harm reduction services and SUD treatment encounter significant work stress and hazards as well [23]. Studies conducted before the COVID-19 pandemic highlighted alarming rates of fatigue and burnout among workers at community-based harm reduction and syringe access programs, especially among workers with lived experience [22, 24, 50, 51]. Harm reduction workers in the United States experience burnout for many of the same reasons that other healthcare workers do, but they also contend with severely under-resourced working environments, a political environment hostile to their work, and low pay, which may all contribute to burnout [24, 52, 53]. Despite calls for increased attention to burnout and precarious work environments in harm reduction spaces, this issue has not been thoroughly studied [52, 54].

### Shanafelt and Noseworthy’s model of key drivers of burnout and engagement

Shanafelt and Noseworthy’s model of key drivers of burnout and engagement provides a framework for understanding factors contributing to and alleviating burnout among physicians and other healthcare workers [55]. This model outlines seven key drivers that impact the spectrum of burnout and engagement: (1) meaning in work, (2) workload and job demands, (3) control and flexibility, (4) work-life integration, (5) social support and community at work, (6) organizational culture and values and (7) efficiency and resources. This model describes the impact of intersecting relationships between individual workers and the organizations at which they work, without placing responsibility for the development or alleviation of burnout on the individual alone [56]. Prior models of burnout place undue burden on the individual worker. This leads to solutions that are only personally, instead of systemically beneficial [55–57]. Likewise, prior models that focus solely on systemic or organizational factors fail to account for individual influence and are unlikely to result in meaningful mitigation of burnout [55, 56]. Shanafelt and Noseworthy’s model stands out as it offers a multi-tiered and holistic view of the complicated and multi-faceted issue of burnout. However, because this model and others like it were designed based on the experiences of workers in healthcare environments like hospitals, it may not fully encompass the experiences of harm reduction and overdose prevention workers, who often work in non-traditional healthcare spaces, like syringe access programs, mobile vans, and on the street. In addition, existing burnout models like Shanafelt and Noseworthy’s focus on physician experiences and may not encompass the full range of volunteer and professional roles that comprise overdose prevention, harm reduction, and SUD treatment. However, because there are currently no existing models focused on burnout among harm reduction, overdose prevention, and SUD treatment workers, it is important to examine how existing models of burnout both reflect and fail to account for the experiences of this vital group of healthcare providers.

In this secondary analysis of a qualitative descriptive dataset, we focused on the phenomenon of burnout among Philadelphia-based harm reduction advocates, community organizers, and SUD treatment clinicians during the COVID-19 pandemic. We explored the COVID-19-related work experiences of Philadelphia-based harm reduction advocates, community organizers, and SUD treatment clinicians and how these experiences mapped onto Shanafelt and Noseworthy’s model of key drivers of burnout and engagement in physicians. We aimed to assess the applicability of this physician-centric model to the experiences of other healthcare workers outside of traditional healthcare settings.

## Methods

### Participants

In the qualitative descriptive parent study from which the data for this secondary analysis was drawn, we interviewed 30 harm reduction advocates, community organizers, and SUD treatment clinicians in Philadelphia during July and August 2020 about their experiences working in their roles during the COVID-19 pandemic [58]. Any individual who identified as working in a paid or volunteer position in harm reduction and/or SUD treatment in Philadelphia was eligible to participate.

### Data collection

After approval was granted from the University of Pennsylvania Institutional Review Board, the principal investigator (PI) of the parent study recruited participants via social media postings and targeted outreach to harm reduction advocates, community organizers, and SUD treatment clinicians known to the study team, and she conducted all interviews. For more information about the PI’s background and training, please see the parent study paper [58]. She also used snowball sampling to recruit additional participants. All participants read the informed consent documents and gave verbal informed consent (as approved by the University of Pennsylvania IRB) prior to interviews. Interviews were semi-structured, one-on-one, lasted approximately 30–45 min, and took place via BlueJeans teleconferencing software or phone based on participant choice. Interviews were audio-recoded and transcribed. The design of the interview guide was based on the Social-Ecological Model [59] as the parent study was focused on how Philadelphia’s harm reduction advocates, community organizers, and SUD treatment clinicians responded to the overdose and homelessness crises during COVID-19, how related policy shifts at local and national levels have impacted their work, and how they believed that the pandemic will affect the future of overdose prevention, harm reduction efforts, and homelessness advocacy in Philadelphia [58]. The interview guide included questions about how participants’ work impacted them personally and their experiences with overwork and burnout. Participants received a $20 VISA gift card as compensation.

### Data analysis

During the first round of analysis for the parent study, two research team members used NVivo 12 software to analyze interview transcripts with thematic analysis methodology [60]. They first read through transcripts to become familiar with the content. They then created a preliminary codebook by coding the first three interviews and used this codebook to code the remaining interviews. They met weekly throughout this process to discuss the analysis and refine the codebook. With input from other research team members, the coders then developed themes based on the codebook.

After this initial analysis, the parent study PI and another research team member conducted a secondary analysis focused on all codes related to overwork and burnout, which had been grouped into a broad theme during the initial analysis. For the present study, two study team members recoded these data deductively, using the Shanafelt and Noseworthy’s model of key drivers of burnout and engagement in physicians to guide this secondary analysis. These two coders met weekly throughout this process to discuss the analysis and settle any disagreements. Codes were then grouped into the seven themes outlined in Shanafelt and Noseworthy model. Study design and analysis were in adherence with Consolidated Criteria for REporting Qualitative (COREQ) checklist guidelines [61].

## Findings

### Participant characteristics

Participants were nurses, physicians, social workers, peer recovery specialists, and volunteer community organizers. Participants came from 15 different organizations. Six participants were volunteers in non-paid positions (community organizers, activists, etc.). Average participant age was 31.6 years (range 16, SD: 6.1), and average number of years of experience working in the present role or similar roles was 5.6 (range 16.5, SD: 5.0). Twenty-two participants (73%) were women, 6 (20%) were men, and 2 (0.6%) were gender non-conforming/non-binary. Additional information about study participants can be found in the parent study [58].

### Results

In alignment with Shanafelt and Noseworthy’s model of key drivers of burnout and engagement, we grouped our codes into seven key themes reflecting the factors associated with burnout included in the model: (1) workload and job demands, (2) meaning in work, (3) control and flexibility, (4) work-life integration, (5) organizational culture and values, (6) efficiency and resources and (7) social support and community at work.

#### Workload and job demands

Participants emphasized that their organizations struggled to accomplish required tasks because of the pandemic. Many participants shared that their workloads were impacted by changing work hours and job locations, being expected to do the same amount of work or more with staff shortages, and, in some cases, adjustments in the scope of their roles. For many participants, working remotely was impossible due to the nature of their organization and the communities they served (i.e., participants working at harm reduction organizations providing syringe access and other resources directly to PWUD). However, some participants reported that organizations tried to limit staff exposure to COVID-19 by asking workers to present in person less frequently. Although some participants acknowledged the benefits of working from home, like decreased COVID exposure risk and a less chaotic environment from which to work, they also discussed how working remotely oftentimes led to an increased amount of work on the days that they were working in person.

For participants who volunteered their time as harm reduction community organizers, efforts to decrease the spread of COVID-19 meant that activities groups used to do together in person were done individually. This shift resulted in increased workloads for some members without the benefit of social support and connection:

“…The way that we put together, construct and prepare [harm reduction] outreach materials has been affected because pre-COVID we were having group events, like kit making parties where people – volunteers would come together and make wound care and works kits in a group, which certainly spread out the labor of doing that quite a bit.” (Harm reduction community organizer, volunteer position)

The changing demands sometimes led to participants’ jobs shifting away from the harm reduction work they previously did. In some cases, workers skilled in using harm reduction frameworks to address substance use felt that their organizations were not supportive of them using these same skills to address their new set of tasks that were altered by the COVID-19 pandemic. One participant described her frustration with her organization’s disinterest in applying a harm reduction framework to her new tasks. In this situation, her position shifted from expanding access to harm reduction supplies to assisting unstably housed individuals move into isolation rooms in “COVID hotels” when they tested positive for COVID. Despite the fact that access to harm reduction supplies was still a need for many of these individuals, this participant did not feel like harm reduction was a priority in her new role:

“And then you were pulled into something that wasn’t really necessarily part of your job, and then it sounds like they weren’t even really interested in you using a harm reduction lens to achieve this. So it’s like they just wanted more hands on deck but they weren’t really utilizing skills.” (Social worker, paid position)

The COVID-19 pandemic caused a reduced workforce, and as a result, the remaining workers were forced to adjust and absorb tasks previously performed by others to make up for labor shortages. Participants described situations of “task shifting,” which has been previously described in studies of harm reduction and SUD workforces [52]. Participants also voiced feeling overwhelmed both by the burden of additional work and perceived lack of sufficient training to perform some of these duties — for example, case managers who had previously worked with clients to secure housing being asked instead provide case management in a buprenorphine program. Some organizations tried to support workers by allowing those who were immunocompromised to work remotely. While participants voiced support for this approach, it often resulted in increased job demands for workers who continued to work in person. One participant described the stress associated with ensuring that necessary work was completed and assuring that individual well-being and equity in the workplace were not compromised:

“…It means that someone else is picking up their work, and how do you also support the person who now has extra work to do? And so things like that have been challenging. I think it’s brought up a lot of ethical dilemmas and highlighted some of the places where there are imbalances and also…injustices.” (Case manager, paid position)

Some participants also expressed equity concerns related to which workers were able to work remotely. Some organizations allowed for prescribing clinicians in SUD treatment programs to work remotely and communicate with patients via telehealth while case managers and peer recovery specialists had to work in person.

Participants clearly described how these changes to workload and job demands led to symptoms of burnout:

Everyone is more stressed out. So I’m more stressed out, my coworkers are more stressed out and the guests are more stressed out…And that’s hard when you’re trying to…navigate your relationships with your coworkers and try to maintain good rapport with guests when you’re also stressed out and less patient than usual…Which means even though we have fewer people, which is easier in some ways, the people we have are more on edge. So there’s still kind of almost the same amount of conflict which is surprising since there’s fewer people. (Social worker, paid position)

#### Meaning in work

Participants shared that the challenges and strain associated with working during the COVID-19 pandemic impacted their ability to find purpose and meaning in their work. In some cases, this was directly related to organizational shortages leading to what participants perceived as a reduction in service quality:

“So I think that the organization has…changed the amount of dignity that we’re able to deliver with our patient interaction, it’s also changed the way that the staff see their roles. They seem to have less of a – less conviction that what we’re doing is absolutely the right thing and they’re more questioning of that…It’s hard not to just pass on whatever oppression that your funding source has on to the patients.” (Social worker, paid position)

Shifting job demands, unpredictability, and overwork had a direct impact on participants’ capacity for emotional engagement with clients/patients. This participant expressed feeling disturbed by her newfound ability to “switch off” empathy in response to overwhelming circumstances over which she felt she had little control:

“I have noticed in myself I have less patience and that patience runs out a lot quicker. Same thing with like empathy. I feel like I have less empathy. And I know it’s not true, but I feel like I could easily switch it off whereas before there was like plenty, plenty of it and that governed my perspective whereas now it’s a lot easier for me like, nope, we can’t do anything, let’s just do it tomorrow.” (Social worker in leadership role, paid position)

A participant working at a low-barrier SUD clinic at a harm reduction center serving some of the most marginalized PWUD in Philadelphia shared how the desperation felt by his patients impacted his outlook and perception of his work:

“The future might not have been bright in the before times, but now it’s dark and chaotic and that has lent a real – I don’t know. It’s colored all of my interactions with the community. That people don’t seem particularly hopeful about the future and that if you’re going to be going into recovery, you need to have some sort of vision of a better future and that’s just not a thing that my patients seem to be seeing right now.” (Physician, paid position)

#### Control and flexibility

Many participants shared they felt lack of control over their work environment and working conditions, which manifested in a variety of ways, including concerns about physical safety. For example, attempts to decrease COVID-19 spread led a harm reduction organization where multiple participants worked to conduct client interactions outside the building. While this alleviated some safety concerns related to COVID-19 exposure, it created a situation where workers had less control over their surroundings. Previously, workers could require that clients exhibiting aggressive behavior leave the building, but once all work took place outside, participants were unable to ask an aggressive client to leave the premises:

“So, when we saw that the person was carrying the knife, we just told our guards, hey, there’s someone here, can you try to get them away from everyone else, so that they are not posing a risk or a threat to the rest of our participants? But that’s all we can really do. We notify [the case manager] so that, if the participant ever passes by again, we can try to set up a meeting or set something up…and kind of understand why the situation went down as it did…” (Case manager, paid position)

For other participants, lack of control was related to the scope of their practice. One participant working at a SUD treatment program was frustrated by her organization’s attempts to recoup lost income by incentivizing overwork:

“And then there were some salary changes so that providers were incentivized to see a high volume of patients, and that became a significant change because it almost created this power dynamic of like everyone’s job now is to make sure we get as many people as possible into the visit. And I have hated it. It’s just it’s not what I signed up for…” (Certified recovery specialist, paid position)

Reflecting the impacts of shifting work demands, as addressed earlier, participants often found themselves performing jobs that had little semblance to what they were originally hired to do. Despite her official job description that focused heavily on harm reduction, one participant struggled to convince her superiors to allow her to perform this work. However, rather than wait for the blessing of her superiors, she maintained some degree of autonomy over her work and how she expended her efforts, allowing her to exert some control over the direction of her work:

“COVID-19 completely changed my job and what I was expected to do. And like no matter how much I tried to bring harm reduction into the conversation, they wouldn’t let it be one. And this was one thing I did a lot in my role. Right? I kind of just took things into my own hands – our own hands – and just made sure we kind of tried to do the best that we could for people while knowing that a lot of times we fell short.” (Social worker, paid position)

On the other hand, some participants found that working through the pandemic allowed them a degree of flexibility due to shifts to telehealth and “work from home” mandates. One participant reported feeling liberated by these changes and enjoyed the new sense of control over his workflow:

“We were driving around in this broke down van without a functional air conditioner, parking on one of the busiest drug corners and trying to do medicine in that space…now that COVID has shut that down and I’ve been able to sit in my really comfy home office and talk to patients on the phone, it’s a more positive experience for me, just for the additional comfort of being able to walk downstairs and make myself a sandwich or sit in the cool, or go up and tend my garden while I talk to a patient” (Physician, paid position)

#### Work-life integration

According to Shanafelt and Noseworthy’s model, work-life integration refers to how work expectations align with a worker’s personal values and priorities and their family and health needs, including the ability to take sick time or medical leave. In the context of this study, “work-life integration” focused mainly on participants’ ability to maintain their personal safety and health while balancing job demands, and physical safety was a top concern for participants. Fear about exposure to COVID-19 was expressed during every interview, and many participants shared that they were constantly attempting to balance their concerns about COVID-19 exposure with their commitment to their work. These fears were especially relevant during these interviews (July & August 2020) as vaccines were not yet available, meaning that personal risk for workers was high. One participant who worked as a supervisor stated:

“It’s been really challenging to think about how to take care of people who work here. It’s a tough time to balance people’s own safety. Like one of the things that came up is people who are immunocompromised themselves or taking care of family members who are immunocompromised, we want to support them. We want to – if it means that at this time they’re not going to come into work, we want to support that decision and we want to take care of them.” (RN, paid position)

Many participants reported that their ability to care for their own physical and emotional safety conflicted with job expectations. Although this phenomenon was certainly heightened by the COVID-19 pandemic, for some participants, this was also true beforehand. Increased job demands and worsening conditions in the neighborhoods where many participants worked created a sense of chaos at work that could easily lead to worker burnout:

“The new normal has become a nonstop crisis and urgency because we’re dealing with people’s lives. So I think that’s a big part of it is just finding ways to set boundaries as an organization. To set boundaries as an individual” (Case manager, paid position)

Some participants expressed the inevitability of the risks to personal safety associated with their jobs, some of which were not directly related to COVID-19 exposure, but rather to threats of violence in the neighborhoods where they worked. In this quote, a participant expresses their view that personal safety risks are “just part of the job,” while also highlighting the belief that only certain types of people are willing and able to do this work. This belief may be responsible for the pressure these workers feel to remain in their positions because of the limited number of individuals prepared to do the work:

“it’s like you just have to make the best decision that you can in that moment, and it might not be safe. And to be honest, even before COVID I felt that way a lot of times sometimes in my job – and you apply COVID to it and it makes it even worse. But being in that neighborhood and being in certain parts of that neighborhood doing certain kinds of work, it’s not always safe. But it doesn’t make it any less important. It just unfortunately takes certain kinds of people to do it” (Case manager, paid position)

#### Organizational culture and values

Many participants expressed a belief that the COVID-19 pandemic had negatively impacted their organization’s culture. Participants shared that they often felt many important decisions were coming “from the top” with little input from workers performing direct service work. In this quote, a participant discusses the decision by leadership at their organization to reduce the number of days that syringe access services were offered:

“…the logic behind that didn’t make that much sense to me. I think that they just felt like they needed to do something to show that we were trying to minimize exposure. But reducing it by two days, it – I don’t know. Nobody who worked within the syringe exchange was really onboard with that, but it was made – a decision made by people in leadership, so.” (Case manager, paid position)

Likewise, another participant lamented what they viewed as leadership’s lack of willingness to improve working conditions despite messaging suggesting that leadership cares about front-line workers:

“it especially sucks during a pandemic, when you’re like, why – y’all are outwardly preaching that we’re a family and are really desperate for people in these sites, staff members in these sites…and yet you’re not actually willing to invest in the things that are necessary to retain people.” (Case manager, paid position)

Due to previous failed attempts to request support or resources from leadership, participants felt resigned to make do with subpar working conditions:

“I’ve only been there for like ten months. But, even still, I feel like my brain is at the point where I’m like…should I really be asking [leadership] for [anything]?…Or not that learned helplessness necessarily, but almost like projecting their response on them because of previous questions or previous advocating and what the response has been.” (Case manager, paid position)

#### Efficiency and resources

Participants in our study spoke about feelings of personal inefficiency at work caused by resource shortages that were exacerbated by the COVID-19 pandemic. Shortages were seen in both the availability of supplies like syringes and treatment capacity at SUD treatment centers in the area generally – treatment centers (like inpatient facilities) where workers were expected to successfully refer their clients. These shortages seriously impacted participants’ ability to do their work:

“But now…we also barely have enough supplies like we did prior, we’re only able to run the syringe service Monday, Tuesday and Friday. And, even at that, it’s at very limited capacity and we definitely had to cut down on the amount of clean syringes and supplies that we are distributing…” (Social worker, paid position)

Another participant stated;

“I think the first thing that came to mind was just resource scarcity…it does feel like a lot of resources are not accessible. So even taking someone to treatment, everything…people take longer in facilities with each individual, they have processes on how to screen people.’ (Case manager, paid position)

#### Social Support and Community at Work

Findings falling under this theme mainly concerned participants’ loss of in-person connection with co-organizers and colleagues due to shifts to remote work and lack of opportunities for socializing (cancelation of work parties, picnics, in-person meetings, etc.):

“We’re not able to have meetings in person either, so all of our organizing has to happen over text message and over Zoom. And that is definitely a different dynamic and a different quality than being able to meet in person and share food together.” (Harm reduction community organizer, volunteer position)

Participants who volunteered as community organizers with harm reduction groups shared that a major aspect of their work involved preparing materials for outreach efforts, which had previously taken place at “kit-making parties.” Although collective members were able to complete these tasks to minimize COVID exposure, the lack of opportunity for socializing was cited as a major loss:

“Well, we did used to gather in groups in order to make the kits that we give out, and that was like a way for the collective to kind of like hang out and do stuff together while also getting work done. We’ve not been able to do that because social distancing.” (Harm reduction community organizer, volunteer position)

We also found that many participants ended interviews thanking the PI for the opportunity to discuss their experiences, with one participant describing the interview as “therapeutic.” This suggests that many participants perhaps lacked an outlet for venting their frustrations about work lives that may be foreign to individuals not working on the front lines in harm reduction and SUD treatment during the COVID-19 pandemic.

## Discussion

This study explored the work-related experiences of Philadelphia-based SUD treatment providers, harm reduction workers, and community organizers during the first few months of the COVID-19 pandemic. Our secondary data analysis of previously collected interview data was guided by a model of physician burnout outlining seven key drivers of burnout and engagement [55]. We aimed to assess the applicability of this physician-centric model to the experiences of other healthcare, social service, and harm reduction providers working outside of traditional healthcare settings. Our study was one of the first to assess burnout among overdose prevention and SUD treatment workers during the early months of the COVID-19 pandemic and is unique in its inclusion of both paid and volunteer workers and workers of different specialties/disciplines.

Our findings fit Shanafelt and Noseworthy’s model; all seven drivers of burnout and engagement were represented in our data. However, we found that participants’ concerns often differed from the examples provided in their model. Shanafelt and Noseworthy’s “work-life integration” driver involves the ability to align work with personal preferences and life demands, including childcare, vacation time, and medical leave. Our participants discussed the alignment of their roles with their personal lives; however, these discussions focused heavily on concerns related to personal safety at work, something not addressed in Shanafelt and Noseworthy’s model. This was likely because exposure to COVID-19 was a pressing concern for our participants at this time as vaccines were not yet available when these interviews took place. Additionally, many participants worked in settings that often felt unsafe, even before the COVID-19 pandemic, due to threats of violence in the neighborhoods where their workplaces were located. The fact that this was not addressed in Shanafelt and Noseworthy’s model reflects the differences in our participants’ work environments compared to traditional healthcare settings. Unfortunately, recent episodes of violence in traditional healthcare settings—including the Tulsa Saint Francis Hospital shooting that killed three healthcare workers [62]—will likely require future models of healthcare worker burnout to consider threats, fears of violence, and concerns about personal safety at work.

Similar to Shanafelt and Noseworthy’s model, indications that the work environment may play a role in the development of burnout among community-based SUD and harm reduction workers were present across themes. While Shanafelt and Noseworthy primarily cite control over scheduling and workhours in this “control and flexibility” driver, our participants shared that they oftentimes felt as though they had little control over their *physical* environments, which sometimes translated into concerns about their physical safety. Unlike traditional healthcare settings like hospitals, harm reduction programs and low-barrier SUD treatment programs may operate out of non-traditional settings such as mobile vans in neighborhoods with large communities of individuals experiencing homelessness and frequent public drug use [63–65]. Operating in these environments allows workers to “meet people where they are” and decrease the barriers associated with receiving care in a traditional clinic or hospital setting [66]. However, the trade-off for workers may be that they have less control over their work environments. Models of burnout that encompass experiences of healthcare workers in non-traditional healthcare spaces must be developed to adequately account for these factors.

Furthermore, Shanafelt and Noseworthy’s model highlights the meaning and satisfaction derived from work. We found this to be salient for participants in our study, who cited deep personal connections to substance use, SUD, and harm reduction. Some participants felt a strong connection to the mission of their work that compelled them to work as unpaid community organizers in addition to their paid jobs. As stated in the model, finding meaning in work was an important protective factor from the development of burnout. It may also lead individuals to stay in jobs that cause significant stress out of a concern that the work will not get done otherwise. As stated by one participant, “*It just…takes certain kinds of people to do it.”* These findings mirror those of Wang et al., who studied the COVID-19 era experiences of syringe service workers in Massachusetts and found that their participants’ lived experience of substance use and/or recovery informed their dedication and a sense of duty to their work [67].

Reflecting the work of Olding et al., our participants cited the impacts of pervasive task-shifting on the development of burnout symptoms [52]. In their study of burnout among SUD peer workers in Vancouver prior to the COVID-19 pandemic, Olding et al. found that task-shifting—defined as reassigning overdose response and other duties from licensed healthcare professionals to workers who do not receive the same training or compensation to perform the work—represented an important driver of burnout and work-related stress [68]. Our participants reported that pandemic-related worker shortages led to the transferring of duties to individuals who remained at their organizations—even if there wasn’t the time or resources to train them properly. For example, some participants reported that case management staff who had no previous experience in a opioid use disorder management program were asked to provide buprenorphine-specific case management due to staffing issues, despite having little training in this area. As highlighted by the work of Olding et al., individuals with lived experience of substance use and SUD bring invaluable knowledge and expertise to SUD treatment and harm reduction work. However, without the credentials of their peers who are licensed healthcare providers, they are also often inadequately compensated and receive insufficient training and support, despite providing the same vital overdose response and case management services.

The overdose mortality rate in Philadelphia continues to rise [13], and harm reduction and SUD treatment efforts still suffer from inadequate funding and insufficient resources. Worker shortages impact the quality of care and the health outcomes of marginalized populations [69] and worsen feelings of burnout among existing workers [56, 57]. Community-based harm reduction work is especially dependent on skilled and compassionate workers from multidisciplinary backgrounds, many with life experiences similar to their clients. Frameworks can help improve understanding of the drivers and mitigators of burnout and guide future research and policy change. However, due to the unique working conditions of harm reduction, overdose prevention, and SUD treatment workers, existing models of healthcare worker burnout may not fully encompass their experiences. Future approaches for addressing burnout among healthcare workers should include those working outside of traditional healthcare spaces and should be specifically targeted to address the needs of harm reduction advocates, community organizers, and SUD treatment clinicians to combat the dual crises of overdose and a lack of resources dedicated to preventing them.

## Limitations

Our study has several limitations. This was a secondary analysis of previously collected data. Although the parent study interview guide contained probes about burnout, the interview guide was not specifically focused on this topic. In addition, data collection occurred over a short period of time relatively early in the COVID-19 pandemic (July–August 2020); additional studies are needed to assess the long-term impacts of the pandemic on overdose prevention and SUD treatment in Philadelphia. Study interviews were conducted via videoconferencing or phone call due to COVID-19 social distancing guidelines; therefore, individuals not able or willing to be interviewed in this way were not eligible to participate limiting the demographics of the individuals we interviewed, etc. Few of our participants worked in leadership positions, and none identified as upper management, so our analysis is lacking this perspective. Finally, our findings may not be reflective of the experiences of harm reduction advocates, community organizers, and SUD treatment clinicians outside of Philadelphia.

## Conclusion

Burnout among healthcare providers has received national attention during the COVID-19 pandemic. Much of this coverage and the existing research have focused on workers in traditional healthcare spaces and often do not consider the experiences of community-based SUD treatment and harm reduction providers. Our findings indicate a gap in existing frameworks for burnout and a need for models that encompass the full range of the harm reduction, overdose prevention, and SUD treatment workforce. As the overdose crisis continues in Philadelphia and across the United States, it is vital that we address and mitigate experiences of burnout among harm reduction advocates, community organizers, and SUD treatment clinicians to protect their wellbeing and to ensure the sustainability of their invaluable work.
